# Multidigit tactile perception I: motion integration benefits for tactile trajectories presented bimanually

**DOI:** 10.1152/jn.00022.2022

**Published:** 2022-07-13

**Authors:** Irena Arslanova, Shinya Takamuku, Hiroaki Gomi, Patrick Haggard

**Affiliations:** ^1^Institute of Cognitive Neuroscience, University College London, London, United Kingdom;; ^2^NTT Communication Science Laboratories, Nippon Telegraph and Telephone Corporation, Atsugi, Japan

**Keywords:** multi-digit, sensory integration, somatosensory, tactile perception, touch perception

## Abstract

Interactions with objects involve simultaneous contact with multiple, not necessarily adjacent, skin regions. Although advances have been made in understanding the capacity to selectively attend to a single tactile element among distracting stimulations, here, we examine how multiple stimulus elements are explicitly integrated into an overall tactile percept. Across four experiments, participants averaged the direction of two simultaneous tactile motion trajectories of varying discrepancy delivered to different fingerpads. Averaging performance differed between within- and between-hands conditions in terms of sensitivity and precision but was unaffected by somatotopic proximity between stimulated fingers. First, precision was greater in between-hand compared with within-hand conditions, demonstrating a bimanual perceptual advantage in multi-touch integration. Second, sensitivity to the average direction was influenced by the discrepancy between individual motion signals, but only for within-hand conditions. Overall, our experiments identify key factors that influence perception of simultaneous tactile events. In particular, we show that multi-touch integration is constrained by hand-specific rather than digit-specific mechanisms.

**NEW & NOTEWORTHY** Object manipulation involves encoding spatially and temporally extended tactile signals, yet most studies emphasize minimal units of tactile perception (e.g., selectivity). Instead, we asked participants to average two tactile motion trajectories delivered simultaneously to two different fingerpads. Our results show strong integration between multiple tactile inputs, but subject to limitations for inputs delivered within a hand. As such, the present study establishes a paradigm for studying unified experience of touch despite distinct stimulus elements.

## Graphical abstract

**Figure:**
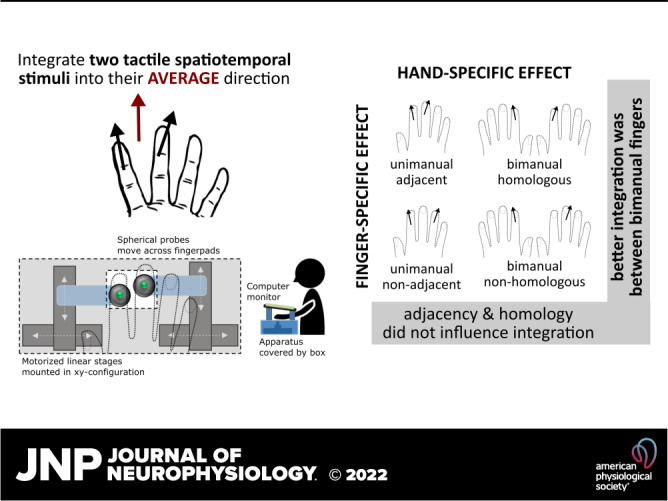

## INTRODUCTION

When an object slips across the skin during manual manipulation, each finger receives distinct information about object’s movement. A unified experience of the object’s motion arises due to the brain’s ability to integrate individual afferent inputs across spatially discontinuous parts of the receptor surface (the skin). Although several studies have revealed mechanisms of tactile motion perception (1–5), these investigated single-digit stimulation. For multi-digit touch, considerable attention has been given to perceptual selectivity, whereby participants need to process a single-stimulus element along a distracting stimulation. These studies have revealed obligatory interactions between competing inputs, such as masking (6, 7) and assimilative biases (8, 9). These interactions typically arise as a function of somatotopic proximity between the inputs, with stronger interactions occurring for somatotopically adjacent skin regions (7, 10, 11). However, everyday tactile experience is not limited to selectively processing one tactile input against another, and often consists of the synthesis of multiple inputs in a unified percept.

Such unified perception, despite distinct inputs, has remained relatively neglected, especially outside of Gestalt perception, where the focus is on how separate inputs are represented as a part of a bigger whole (12), and multisensory integration, where the focus is on integrating redundant sensory cues (13, 14). The multi-touch integration, we are interested in, best resembles the tradition of ensemble perception in vision (15, 16), whereby an observer can represent the overall *gist* information across multiple regions of the sensory surface, each of which may individually receive varying stimulation. Consider perceiving the overall movement of a flock of birds or sensing the overall movement of rice as you wash it by hand. In vision, it has been shown that participants can discern the average motion from simultaneous discrepant motion cues (17, 18)—here we characterize an equivalent process in touch.

A handful of studies have investigated multi-digit integration of frequency- and intensity-based signals (8, 19–21). For example, Walsh et al. (20) and Cataldo et al. (21) asked participants to judge the overall intensity of two electrical shocks of different intensities delivered concurrently to two different fingers. Interestingly, they found that when the discrepant stimulus pairs were delivered to the fingers of the same hand, participants’ judgments were biased toward the more intense stimulus. Importantly, the weaker stimulus was not completely extinguished and still contributed to the ultimate percept, indicating a mechanism that attempts to aggregate multiple inputs, but does so in a manner biased toward the stronger stimulus. This bias was not apparent when the same stimuli were applied to the fingers on the opposite hands.

There are two potential explanations for this finding. First, interactions between tactile inputs mentioned earlier, which are stronger for adjacent skin regions, could lead to biased aggregated final percept, but do not extend across the two hands. Second, limited capacity to divide attention and process multiple stimuli in parallel could lead to biased ensemble perception. Tactile perception is known to have strict capacity limits (22–25). For example, Gallace et al. (23) found that when people need to count simultaneous brief tactile stimuli distributed across the body surface, the error rates increase rapidly (>30%) whenever more than two tactile stimuli are presented at once. Notably, such capacity limit may be relieved when tactile stimuli are delivered to opposite sides of the body (26–28).

In our previous study (29), we found evidence for a neural mechanism that promotes multidigit tactile motion integration by modulating the degree of interactions between stimulated digit representations, but we did not manipulate somatotopic distance between the stimulated digits. Here, we extended our findings by characterizing multidigit spatiotemporal integration in terms of somatotopic distance and stimulus discrepancy. Specifically, in *experiments 1* and *3*, the stimuli were delivered to fingers on the same hand, whereas in *experiments 2* and *4*, the stimulated fingers were on different hands. In addition, in *experiments 3* and *4* we further manipulated the somatotopic relationship (adjacency and homology) between stimulated fingers. We used single-point stimuli that produced a continuous displacement across the skin ensuring a successive stimulation of afferent fibers in a systematic spatial path (see Refs. 30–32 for similar stimuli). Although, different types of stimuli have been used to characterize tactile motion perception, such as dense Braille-like arrays (1–3, 33) and rotating drum-like stimuli (34–36), our main imperative for using single-point moving stimuli is the ease of creating distinct motion paths across nonadjacent skin regions and the fine-grained control over the properties of the concurrent stimulus trajectories, such as their motion discrepancy.

## METHODS

### Participants

In total, 60 participants took part in the study, 15 in each of four experiments. All participants gave written informed consent before experiments, in accordance with the declaration of Helsinki. The study was approved by the University College London Research Ethics Committee. Seven participants were excluded from analysis (4 in *experiment 1*; 1 in *experiment 2*; 1 in *experiment 3*; 1 in *experiment 4*), because they had estimation errors exceeding 20° in more than 50% of trials in at least one single-finger condition, making their data unreliable. Excluded participants were replaced with others. The demographics of the final sample were as follows: *experiment 1* (age range: 21–39; mean: 27.07; 10 women, 5 men; all but one reportedly right-handed); *experiment 2* (age range: 22–40; mean: 25.87; 9 women, 6 men; all reportedly right-handed); *experiment 3* (age range: 18–30; mean: 23.47; 11 women, 4 men; all but one reportedly right-handed); *experiment 4* (age range: 18–34; mean: 22.20; 10 women, 5 men; all reportedly right-handed). A sample size of 15 was estimated using G*Power 3.1 (37), based on desired power of 0.80 and an effect size of 0.68 for aggregated versus single-digit motion perception precision (unpublished pilot study).

### Tactile Apparatus

**Figure 1.**
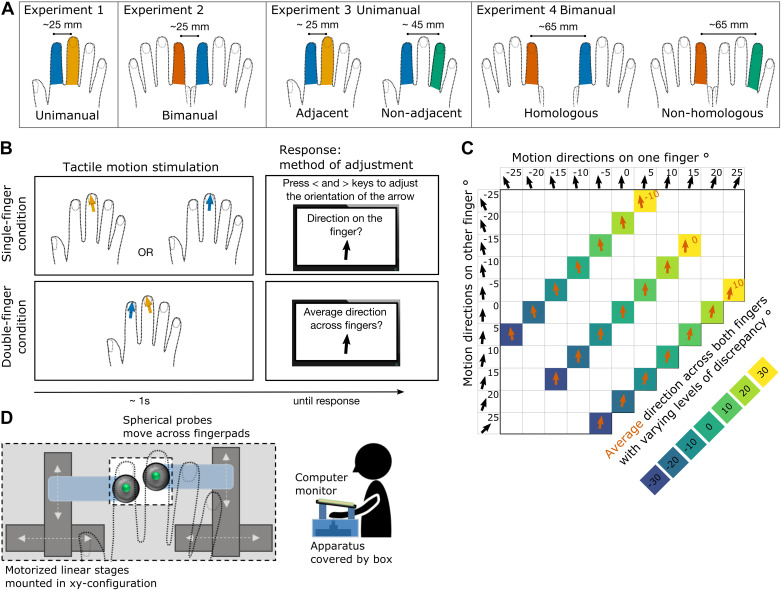
Methods. *A*: stimulated fingers in each experiment and condition. *B*: paradigm and trial example from *experiment 1*. Fingers were stimulated in two main conditions: single-finger condition and double-finger condition. In single-finger condition, in different blocks, just the index or just the middle finger was stimulated. Participants reported the direction of the stimulus’ movement across their fingerpad. To do so, they adjusted a visual pointer on the screen after each trial (by pressing left and right keys or left and right foot pedals). In double-finger condition, both component fingers were stimulated simultaneously, and participants had to judge the average direction between the two component directions. *C*: tactile stimuli. Eleven component single-finger directions were combined into 3 average directions with 7 levels of discrepancy. The sign of discrepancy reflects whether the component directions tended to converge (negative discrepancy) toward the inner edges of fingertips or diverge (positive discrepancy) toward the outer edges of fingertips. *D*: tactile motion apparatus and setup. Tactile apparatus consisted of two spherical probes (4 mm diameter) attached to two motorized linear stages mounted in a *XY*-axis configuration. The apparatus was covered by a wooden box with a rectangular gap, which was used to guide finger placement and secure the hand position. A computer screen, where participants indicated their response, was placed above the hand.

It consisted of two spherical probes (4-mm diameter) attached to two stepper linear actuators (Haydon Kerk Motion Solutions 15000 series, model LC1574W-04) that were fixed to two motorized linear stages (Zaber X-LSM100B, Zaber Technologies Inc., Canada) mounted in an XY configuration (Fig. 1*D*). The actuators were controlled by a microcontroller (Arduino) and were moved vertically so that a plastic hemispheric probe (diameter 4 mm) could make/break static contact with the fingerpad skin at the start of tactile stimulation and retract after the end of stimulation. The probe could be moved horizontally in predefined trajectories (see *Tactile* S*timuli*). The apparatus was covered by a box with a small aperture. To-be-stimulated fingertips were positioned in a palm-down position over the aperture and secured with foam padding. During the stimulation the probes lightly touched the fingerpads. A webcam was placed under the apparatus to monitor the finger placement and contact with the probe. The aperture was then covered with a screen, so that participants could not see the stimuli. The same apparatus was used for all experiments.

### Tactile Stimuli

Continuous motion along the fingertips was created by moving the probes at preselected angles ranging from −25° to 25° to the distal-proximal finger axis in 5° steps, at a constant speed of 10 mm/s. The movement of each probe was controlled individually allowing for delivery of trajectories with varying discrepancy simultaneously along both fingertips. Figure 1*C* shows 21 possible combinations compromised of 11 individual directions delivered simultaneously to two fingers. The combinations produced three different average motion patterns, with varying discrepancy between the two stimuli. The sign of discrepancy reflects whether the component directions tended to converge toward the inner edges of fingertips (negative discrepancy) or diverge toward the outer edges of fingertips (positive discrepancy). At the beginning of each trial, the probe was advanced to make a static contact with the fingertip. The initial position of each probe was jittered across trials (−2.0, 0.0, or 2.0 mm from the center of the fingertip) to discourage using memory for locations as a proxy for direction. After each trajectory, the probe was immediately retracted and returned to its starting position. The duration of each trajectory was ∼1 s and the distance traveled was 10 mm.

### Experimental Design and Procedure

In all conditions, participants rested their hand(s) in a fixed palm-down position (Fig. 1*D*). In *experiment 1*, the probes, through the aperture, lightly touched the center of their right index and middle fingertips. In *experiment 2*, the probes touched the right index and left index fingertips. In *experiment 3*, the setup was identical to *experiment 1*, and additionally included a nonadjacent condition, in which the probes touched the right index and right ring fingers. In *experiment 4*, the setup was identical to *experiment 2*, and additionally included a nonhomologous condition, in which the probes touched the left index and right ring fingers (Fig. 1*A*). The distance between fingers and corresponding width between the probes was held fixed across conditions whenever possible to minimize the effects of spatial distance between fingers. Thus, in *experiments 1* and *2*, the distance between the fingers was fixed to ∼25 mm. In *experiment 3*, the fingers in the adjacent condition remained at ∼25 mm; in the nonadjacent condition, the distance between the index and the ring finger was ∼45 mm. In *experiment 4*, the distance between the bimanual fingers was fixed to ∼65 mm to make the homologous and the nonhomologous conditions comparable without the confounding effects of spatial separation.

To investigate perception of the average motion pattern from two separate trajectories, we compared perception of the average direction to perception of the two individual component stimuli presented alone (Fig. 1*B*). Accordingly, all experiments contrasted double-finger stimulations with the single-finger stimulations of which they were composed. Single-finger conditions were repeated for each finger (e.g., in *experiment 1* participants performed estimation on index finger and separately on middle finger). The single-finger conditions for each finger were then averaged to obtain one measure characterizing single-finger perception. In all conditions, participants gave their response after the motion stimuli ended, by adjusting the orientation of a visual arrow that appeared on the computer screen placed immediately above their fingertips. They had to adjust the arrow’s orientation to the perceived single-finger direction (single-finger condition) or average direction (double-finger condition). The adjustment was made either by pressing left and right arrow keys (and enter key to record their final response) with their (unstimulated) left hand in *experiments 1* and *3*, or by pressing anticlockwise and clockwise rotator pedals with the left foot (and a third pedal with the right foot to record their response) in *experiments 2* and *4*. Responses were unspeeded, and no feedback was given. After the response, the arrow disappeared, and the probes moved to their starting positions.

In *experiments 3* and *4*, we further examined how averaging ability changes as a function of somatotopic distance between fingers. We compared the contrast between estimating individual directions (single-finger condition) and estimating their average (double-finger condition) across two different somatotopic distances. In *experiment 3*, the fingers were either adjacent (right index and right middle) or nonadjacent (right index and right ring). In *experiment 4*, the fingers stimulated were either bimanually homologous (right index and left index) or nonhomologous (right ring, left index).

All conditions were blocked. In *experiments 1* and *2*, the order of finger conditions was counterbalanced across participants. In *experiments 3* and *4*, the order of somatotopy conditions (adjacency or homology) was counterbalanced across participants, and finger condition was counterbalanced within each somatotopy condition. In double-finger conditions, the 21 stimulus combinations were repeated seven times in *experiment 1* and *experiment 2*, five times in *experiment 3*, and six times in *experiment 4*. In single-finger conditions, the number of repetitions of each direction was matched to that in the double-finger condition. The total number of trials was 168 per condition in *experiment 1* and *experiment 2*, 105 per condition in *experiment 3*, and 120 per condition in *experiment 4*. *Experiments 1* and *2* lasted ∼1.5 h, whereas *experiments 3* and *4* lasted ∼2 h and were performed across two 1-h sessions on separate days.

### Analysis of Main Measures: Sensitivity, Bias, and Precision

**Figure 2.**
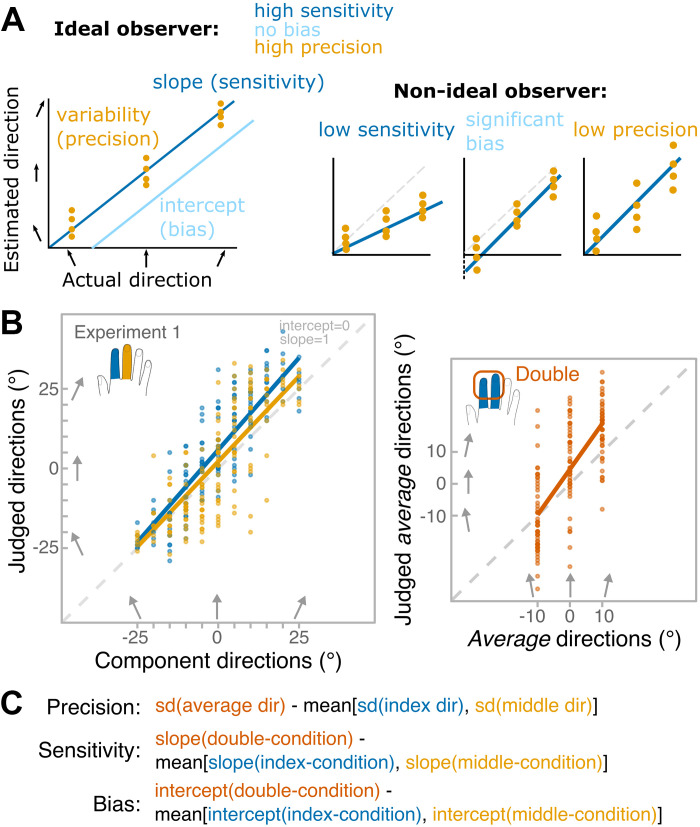
Analysis. *A*: measures used to characterize tactile motion perception: sensitivity, bias, and precision. Sensitivity quantifies the ability to perceive differences between motion directions. Bias reflects the shift of the perceived 0° motion, in our case corresponding to the midline of the finger. Precision measures the consistency of a direction percept elicited by repetitions of the same stimuli. The schematic panels show that these three measures are independent. *B*: data from an example participant in *experiment 1*. Linear regression was fit to the data separately for the two single-finger conditions (*right*) and the double-finger condition (*left*). Slope and intercept values reflecting sensitivity and bias, respectively, were estimated from the regression fit. The dots indicate repeated judgments of each direction. Standard deviations (SDs) were calculated for each direction reflecting the precision. Note that each average direction was composed of one component direction delivered to the index finger and another delivered to the middle finger resulting in 21 combinations: 7 discrepancy levels for a given average direction. In the *left* plot, the discrepancy has been pooled together. *C*: calculation of main measures. We were interested in the contrast between perceiving the average direction versus estimating individual component directions. For precision we contrasted the SD for average direction to the mean SDs for corresponding component directions, while for sensitivity and bias, we contrasted slope and intercept values of double-finger condition to the mean slope and intercept value of single-finger conditions.

Tactile motion processing was characterized in terms of sensitivity to changes in stimulus direction, bias of direction judgments, and precision of repeated estimates of direction for the same stimulus (Fig. 2). Sensitivity and bias corresponded to slope and intercept values that were estimated by fitting linear regressions to each participant’s data separately in each condition. Group-level adjusted *r*^2^ for linear fits for single-finger conditions were as follows: 0.61 ± 0.07 in *experiment 1*, 0.57 ± 0.11 in *experiment 2*, 0.65 ± 0.09 in *experiment 3* (adjacent), 0.62 ± 0.09 in *experiment 3* (nonadjacent), 0.57 ± 0.15 in *experiment 4* (homologous), and 0.55 ± 0.17 in *experiment 4* (nonhomologous). For double-finger conditions: 0.28 ± 0.12 in *experiment 1*, 0.42 ± 0.11 in *experiment 2*, 0.34 ± 0.11 in *experiment 3* (adjacent), 0.28 ± 0.13 in *experiment 3* (non-adjacent), 0.42 ± 0.17 in *experiment 4* (homologous), and 0.37 ± 0.18 in *experiment 4* (nonhomologous). Figure 2*B* shows the fitted linear regression to a representative participant in *experiment 1*. The slope of the regression reflects the relationship between perceived direction and actual tactile direction, while the intercept reflects the perceptual shift of the perceived midline of a given finger. We expected the slope to be greater than 0 and close to 1, showing that participants’ perception matched the linear change in the probes’ direction. Because we did not manipulate finger posture, and always aligned the finger long axis with the 0° stimulus direction, we expected the intercept to be close to 0 reflecting no general bias in direction judgments (whether directions are perceived more leftward or rightward than they are). The benefit of analyzing slope and intercept values independently rather than calculating the error between actual and judged angle is that slope allows one to characterize perception (whether perception follows the linear change in directions) independent of directional biases (whether directions are perceived more leftward or rightward than they are). When errors were computed instead, they largely mapped onto the intercept values.

A perceiver with a given level of sensitivity or bias may be more or less precise (Fig. 2*A*). We used unbiased standard deviation (SD) of repeated judgments of the same stimuli as a measure of precision. Unbiased SDs were measured over repeated different tactile motion stimuli. In other words, in single-finger conditions, SDs were measured for each direction. We took the mean of these values to reflect the inverse precision for single-finger condition. In double-finger condition, SDs were measured for each combination. The mean of this value reflects inverse precision for double-finger condition. Because we wanted to keep the stimuli in single-finger conditions identical to the ones in double-finger conditions, the number of trials for each single-finger angle varied (i.e., 0° was used 28 times, whereas 25° only 7 times) to match the number of times each was used in double-finger combinations. However, the normal sample SD is a biased estimate of the population SD—the smaller the sample size the more likely it will underestimate the population SD (see section 7.3 of Ref. 38). Therefore, to account for different number of trials in each angle, we used an unbiased SD to calculate precision over angles and combinations with the following equation:

(*1*) $${\text{σ}}_{\text{unbiased}}=\sqrt{\frac{n-1}{2}}\frac{\text{Γ}(\frac{n-1}{2})}{\text{Γ}(\frac{n}{2})}s,$$

where Γ(·) is the γ function and *s* represents the usual standard deviation.

We first analyzed perception of spatiotemporal directions on each single-finger condition. Perceiving the average direction requires participants to integrate the two single-finger component directions. We therefore compared performance for perceiving the average direction with the mean perceptual performance when perceiving the direction of a single stimulus in the single-finger conditions (Fig. 2*C*). Better performance in the double-finger condition would imply a benefit of integration, relative to the performance in the corresponding single-finger conditions. The reason we used a linear regression approach rather than looking at response distributions for each stimulus combinations separately (i.e., average direction 10° with discrepancy of 20° vs. direction 20° on one finger and 0° on the other) was because it provided us with a unified measure for single- and double-finger conditions. However, we need to acknowledge that a criticism can be made in using a linear regression approach to compare the performance in the single-finger conditions to that of the double-finger condition, as in the former the fitting is made against 11 stimulus directions, whereas in the latter it is made against three average directions (see Fig. 2*B*). To avoid this problem, instead of fitting a linear regression, performance can be categorized in terms of errors (i.e., deviation between the judged direction and the actual direction). However, such error-based analysis produced largely comparable results to those contrasting the intercept values between single-finger and double-finger conditions. Thus, we did not report the error-based analyses here.

In all experiments, we first used one-sample *t* tests on slope and intercept values to see whether participants could perceive probes’ directions and whether their perception was unbiased. When normality was violated, sign tests were used instead. In *experiments 1* and *2*, main interest was whether the aggregation process differed if component directions were combined within- or between-hands. We carried out a mixed ANOVA with a within-subjects factor number of fingers (single- vs. double-finger condition) and a between-subject factor experiment (*experiment 1*: unimanual vs. *experiment 2*: bimanual) on sensitivity (slope), bias (intercept), and precision (unbiased SD). In *experiment 3*, the main interest was whether within-hand averaging ability depended on the somatotopic distance between fingers. A repeated-measures ANOVA was used with factors number of fingers (single- vs. double-finger condition) and adjacency (adjacent vs. nonadjacent fingers) on the three measures. In *experiment 4*, the main interest was whether the putative somatotopic mechanism extends to between-hands averaging. Here, similarly to *experiment 3*, repeated-measures ANOVA were used with factors number of fingers (single-finger vs. double-finger condition) and homology (homologous vs. nonhomologous fingers).

For linear models such as ANOVA, normality assumption should be checked not against raw dependent variable, but on the residuals (or errors) from the fitted model (39). There were a few violations of normality at the conventional 0.05 probability level as measured with Shapiro–Wilk test. However, ANOVA with a balanced design is considered robust even with normality violations, thus, violations of parametric assumptions were unlikely to majorly influence Type I and Type II errors. In addition, because our inferences were mainly based on interactions, alternative nonparametric Friedman test would have not been suitable. For these reasons, parametric ANOVA was used throughout.

In addition to the main analyses, we explored whether averaging ability varied as a function of discrepancy between the component directions. Therefore, we fit separate linear regressions to the double-finger data for each discrepancy level and extracted the slopes and the intercepts for each discrepancy. Discrepancy of 0, when component directions were identical, was excluded from these analyses, as those stimuli did not have two signs of direction. For precision, unbiased SDs were calculated for each pair of stimuli (3 average directions by 6 levels of discrepancy). For *experiments 1* and *2*, mixed ANOVA with within-subjects factors sign of discrepancy (diverging vs. converging) and level of discrepancy (30° vs. 20° vs. 10°) and a between-subjects factor experiment (unimanual vs. bimanual) was carried out. For *experiment 3*, repeated-measures ANOVA was used with factors sign of discrepancy (diverging vs. converging), level of discrepancy (30° vs. 20° vs. 10°), and adjacency (adjacent vs. nonadjacent fingers). For *experiment 4*, the same analysis was carried out, except adjacency factor was replaced with homology (homologous vs. nonhomologous fingers). When modeling precision, average direction was added as an additional within-subject factor to reveal any direction-specific differences.

Finally, support for the null hypothesis could be scientifically informative. Because some of our interpretations rely on the absence of an effect, particularly the effects of adjacency and homology in *experiments 3* and *4*, we used Bayes factors (38, 40–43) to assess support for the null hypotheses, in appropriate cases. BF^01^ was used to indicate the strength of evidence for the absence of an effect/interaction over a model that did not contain that specific effect/interaction, while BF_inclusion_ was used to reflect the strength of evidence for including a particular effect/interaction against all other models. We considered BF > 3 and BF < 0.33 showing sufficient evidence, while BF between 0.33 and 3 showing inconclusive evidence (44, 45).

The data used for all experiments, the main estimated measures, and analysis scripts are openly accessible on Open Science Framework (https://osf.io/26ng3/?view_only=025924e13fbb4cf8b75da20d1b9709ae).

## RESULTS

### Single-Finger Conditions

**Figure 3.**
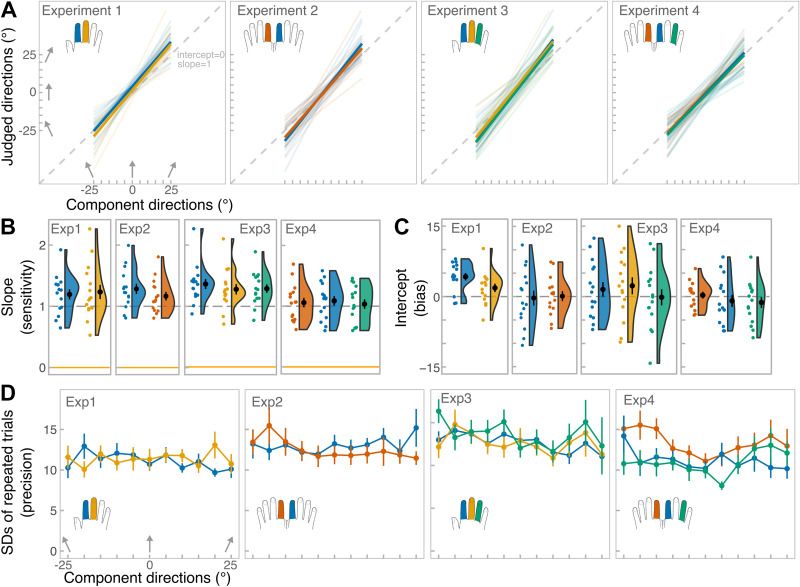
Results for single-finger conditions. *A*: individual fitted linear regressions (transparent lines) and group-level regressions (thick lines) for each finger, separately for each experiment. The gray dashed line represents equality line of slope 1 and intercept 0. *B*: individual slope values (colored dots) together with the group-level means (black dot). Raincloud plots (46) represent data distribution and error bars represent standard error from the mean. Colors on the plots correspond to the finger-conditions in *A*. *C*: individual intercept values together with the group-level means. *D*: group-level standard deviations for repeated trials for each component direction. Error bars represent standard error from the mean.

Single-finger conditions were analyzed first to establish whether participants were able to perceive component directions and whether perception was similar across individual fingers. Figure 3*A* shows individual linear regressions fit for each single-finger condition separately in each experiment, while Table 1 shows the summary of all measures. Note that the focus of the studies was to characterize averaging performance. Thus, while we report all the results for transparency, we only briefly discuss those where necessary.

**Table 1.** Main measures for single-finger conditions

	Slope (Sensitivity)			Intercept (Bias)		SD (Precision)
Condition	Means ± SD	*t* Test Against 0: *t*(*d*)	*t* Test Against 1: *t*(*d*)	Means ± SD	*t* Test Against 0: *t*(*d*)	Means ± SD
*Experiment 1*:						
Index	1.2 ± 0.3	14.39 (3.72)**	2.35 (0.61)*	4.22 ± 3.1	5.35 (1.38)**	11.1 ± 4.1
Middle	1.2 ± 0.5	10.66 (2.75)**	2.02 (0.52)	1.84 ± 3.5	2.04 (0.53)	11.4 ± 4.5
*Experiment 2*:						
Left index	1.3 ± 0.3	16.70 (4.31)**	2.37 (61)*	0.09 ± 4.1	0.08 (0.02)	13.0 ± 5.1
Right index	1.2 ± 0.3	15.43 (3.98)**	3.42 (0.88)*	−0.3 ± 6.2	−0.20 (−0.05)	12.5 ± 5.5
*Experiment 3*:						
Index	1.4 ± 0.3	120† (4.32)**	118† (1.15)**	1.5 ± 6.1	0.97 (0.25)	12.7 ± 4.5
Middle	1.3 ± 0.3	15.02 (3.88)**	3.28 (0.85)*	2.3 ± 7.0	1.26 (0.33)	12.6 ± 6.1
Ring	1.3 ± 0.3	16.26 (4.20)**	4.11 (1.06)*	−0.2 ± 6.6	−0.09 (−0.02)	13.6 ± 6.9
*Experiment 4*:						
Left index	1.1 ± 0.3	13.49 (3.5)**	0.75 (0.19)	0.3 ± 2.8	0.41 (0.11)	13.3 ± 5.9
Right index	1.1 ± 0.3	13.91 (3.6)**	1.12 (0.29)	−0.9 ± 5.0	−0.72 (−0.19)	11.2 ± 5.3
Right ring	1.0 ± 0.3	13.16 (3.4)**	0.45 (0.12)	−1.3 ± 4.5	−1.09 (−0.28)	10.9 ± 5.3

Same group of 15 participants performed under all single-finger conditions within a single experiment, but each experiment had a new group of 15 participants.

* *P* < 0.05; **P < 0.001; † the sign test (V), which was used instead of *t* test, due to non-normality.

Figure 3*B* shows single-subject and group-level slope values for each finger. The slopes (corresponding to sensitivity)were significantly above 0 in all experiments indicating that the component directions could be successfully perceived (performance followed the linear change in the tilt of probe’s direction). In fact, for many conditions slopes exceeded 1, indicating that participants tended to overestimate the divergence of the stimuli from the principal axis of the finger (0°). In *experiment 1*, the slope did not differ between the index and the middle fingers (paired-sample *t* test: *t*_14_ = −0.64, *P* = 0.53, *d* = −0.17). In *experiment 2*, the slope values were slightly higher on the right index compared with left index finger, but the difference was not statistically significant (*t*_14_ = 1.89 *P* = 0.08, *d* = 0.49). In *experiment 3*, one-way repeated-measures ANOVA yielded a marginally significant effect of finger (*F*_2, 28 _= 3.33, *P* = 0.05, $\eta_{\text{p}}^{2}$ = 0.19). We expected perception to be possibly worse on the ring finger compared with the index and the middle fingers. However, planned contrasts showed that ring finger did not differ from the index and the middle fingers (*t*_28_ = 1.03, *P* = 0.31). Rather, the slope for the index finger judgments was slightly higher than for the middle finger judgments (*t*_28_ = 2.37, *P* = 0.03). Yet, the effect was marginal, and all fingers yielded slope values greater than 1. Finally, in *experiment 4*, there was no significant effect of finger (*F*_2, 28_ = 0.54, *P* = 0.59, $\eta_{\text{p}}^{2}$ = 0.14).

Figure 3*C* shows single-subject and group-level intercept values (corresponding to bias). Except for the intercept for the index finger in *experiment 1*, all other intercepts did not differ from 0, indicating no bias. Judgments on index finger in *experiment 1* showed a positive bias of ∼4°, meaning that the perceived midline of the finger was shifted slightly rightward. Indeed, there was a marginal significant difference between single-finger intercepts in *experiment 1* (sign test due to non-normality: *V=* 99, *P* = 0.03, *d* = 0.66). There was no finger difference in *experiment 2* (paired-sample *t* test: *t*_14_ = −0.19, *P* = 0.85, *d* = −0.05). Similarly, there was no effect of finger in *experiment 3* (*F*_2, 28 _= 1.50, *P* = 0.24, $\eta_{\text{p}}^{2}$ = 0.10) or *experiment 4* (*F*_2, 28_ = 0.71, *P* = 0.50, $\eta_{\text{p}}^{2}$ = 0.05).

Figure 3*D* shows unbiased SDs for each component direction (corresponding to inverse precision). A repeated-measures ANOVA with factors finger (stimulated fingers in the experiment), overall direction (leftward vs. rightward) and specific angle (25°, 20°, 15°, 10°, and 5°) was conducted. Component direction 0° was excluded from this analysis, as it could not be divided into leftward versus rightward direction. In *experiment 1*, there was no significant effect of finger (*F*_1, 14_ = 0.18, *P* = 0.68, $\eta_{\text{p}}^{2}$ = 0.01). The effects of overall direction and specific direction were also nonsignificant (*F*_1, 14_ = 0.31, *P* = 0.58, $\eta_{\text{p}}^{2}$ = 0.02 and *F*_4, 56_ = 0.43, *P* = 0.78, $\eta_{\text{p}}^{2}$ = 0.02, respectively). All interactions were nonsignificant as well (*P* values: 0.16 > *P* > 0.90). No effect of finger (*F*_1, 14_ = 0.27, *P* = 0.61, $\eta_{\text{p}}^{2}$ = 0.02) was revealed in *experiment 2*. Other main effects and interactions also remained nonsignificant (all other *P* values: 0.08 > *P* > 0.79). In *experiment 3*, effect of finger was nonsignificant (*F*_2, 28 _= 1.53, *P* = 0.23, $\eta_{\text{p}}^{2}$ = 0.10), but there was a significant effect of overall direction (*F*_1, 14 _= 9.29, *P* = 0.009, $\eta_{\text{p}}^{2}$ = 0.40) with participants being more precise judging leftward (means SD = 12) relative to rightward (means SD = 14) directions. Finally, in *experiment 4*, a significant effect of finger was observed (*F*_2, 28 _= 6.1, *P* = 0.007, $\eta_{\text{p}}^{2}$ = 0.30) with judgments from left index finger being more variable (means SD = 13) than both right index (means SD = 11) and right ring (means SD = 11). In addition, the effect of specific angle was also significant (*F*_4, 56 _= 3.1, *P* = 0.015, $\eta_{\text{p}}^{2}$ = 0.18—Greenhouse-corrected *P* value), but Bonferroni-corrected pairwise tests indicated a significant difference only between 5° and 15° as well as 5° and 25° with judgements of 5° being more precise (means SD = 11) than for 15° (means SD = 12) and for 25° (means SD= 12). Note that 0° was excluded from analysis, but its means SD was also low (means SD = 10). This suggests that precision might have been higher for directions that were around the midline of the finger relative to the ones that deviated away from the midline. But this effect was only present in *experiment 4*.

**Figure 4.**
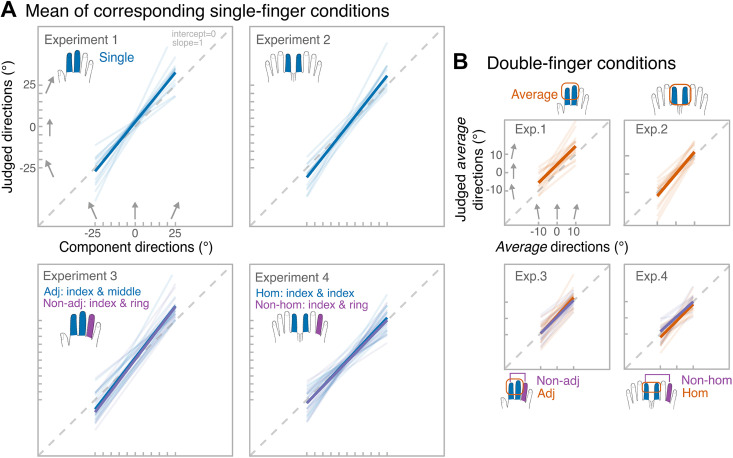
Individual fitted linear regressions (transparent lines) and group-level regressions (thick lines) for each experiment, separately for single-finger conditions (*A*) and double-finger conditions (*B*). The gray dashed line represents the equality line of slope 1 and intercept 0.

In summary, perception across different fingers was similar with few exceptions: increased bias on the index finger in *experiment 1* and reduced precision on the left finger in *experiment 4*. Analysis of the SDs showed that there were few direction-specific effects, namely in *experiments 3* and *4*, however, overall, the direction-related differences were small. Now that the single-finger perception was examined, we moved to our main focus—characterizing the aggregation capacity and whether it is influenced by factors like somatotopic relationship between stimulated fingers and the angular discrepancy between the components. To do this, the single-finger measures were averaged (single-finger condition) and compared with the perception of their aggregate direction (double-finger condition). We try to report all effects for the three measures—sensitivity, bias, precision—for transparency, but we focus our discussion on the effects that we have deemed important. Figure 4 shows the fitted linear regressions in each experiment and conditions that were used to derive the slope and the intercept values. Table 2 shows the overview of the main measures. In all experiments, participants showed slope values that were significantly higher than 0 and close to 1. Interestingly, in single-finger conditions the slope was often above 1. Except in *experiment 1* and in the homologous condition of *experiment 4*, perception was generally unbiased.

**Table 2.** Overview of the main measures across all four experiments

Condition	Slope (Sensitivity)			Intercept (Bias)		SD (Precision)
	Means ± SD	*t* Test Against 0: *t*(*d*)	*t* Test Against 1: *t*(*d*)	Means ± SD	*t* Test Against 0: *t*(*d*)	Means ± SD
Experiment 1:						
Single	1.2 ± 0.4	12.64 (3.26)**	2.24 (0.58)*	3.03 ± 2.8	4.27 (1.10)*	11.2 ± 2.1
Double	1.0 ± 0.4	9.76 (2.52)**	−0.1 (−0.00)	4.42 ± 4.4	3.92 (1.01)*	12.5 ± 3.4
Experiment 2:						
Single	1.2 ± 0.3	120† (4.51)**	108† (0.83)*	−0.12 ± 3.3	−0.14 (−0.04)	12.8 ± 2.4
Double	1.2 ± 0.4	13.09 (3.38)**	2.19 (0.57)	0.08 ± 4.1	0.07 (0.02)	10.9 ± 2.5
Experiment 3:						
A. S	1.3 ± 0.3	120† (4.17)**	116† (0.99)**	1.9 ± 5.9	1.23 (0.32)	12.4 ± 3.5
A. D	1.1 ± 0.4	11.81 (3.05)**	1.14 (0.29)	1.7 ± 5.2	1.26 (0.33)	11.5 ± 3.2
N. S	1.3 ± 0.3	17.66 (4.56)**	4.45 (1.15)*	0.72 ± 6.3	0.44 (0.11)	13.0 ± 3.2
N. D	1.0 ± 0.3	13.75 (3.55)**	0.23 (0.06)	0.95 ± 4.6	0.81 (0.21)	12.7 ± 2.8
Experiment 4:						
H. S	1.1 ± 0.3	14.39 (3.72)**	1.03 (0.27)	−0.2 ± 3.0	−0.27 (−0.07)	11.5 ± 3.2
H. D	1.0 ± 0.4	10.15 (2.62)**	0.22 (0.06)	−1.8 ± 2.8	−2.48 (−0.64)*	9.7 ± 3.5
N. S	1.0 ± 0.3	13.37 (3.45)**	0.88 (0.14)	−0.6 ± 3.2	−0.70 (−0.18)	12.1 ± 4.3
N. D	0.9 ± 0.4	8.54 (2.21)**	−0.76 (0.20)	0.7 ± 3.0	0.95 (0.25)	10.3 ± 4.5

Each condition contains *n* = 15; different groups of 15 participants performed in each experiment. A. S, adjacent single; A. D, adjacent double; N. S, non-adjacent single; N. D, non-adjacent double; H, homologous.

* *P* < 0.05; **P < 0.001; † the sign test (V), which was used instead of *t* test, due to non-normality.

### *Experiments 1* and *2*

**Figure 5.**
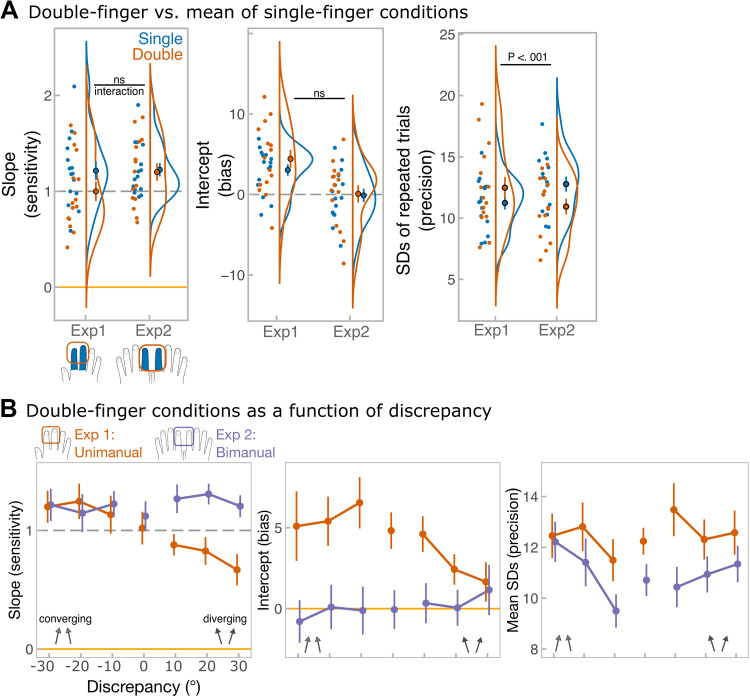
Results of *experiments 1* (unimanual) and *2* (bimanual). *A*: main measures, from the *left*: *1*) slope values, corresponding to the sensitivity, estimated from single-subject regressions; *2*) intercept values, corresponding to the bias, estimated from single-subject regressions; *3*) unbiased SD values for repeated estimation of identical directions. SDs were calculated separately for each of 21 stimulus combinations; for simplicity, data were pooled across combinations to show the means SDs for single-finger (blue) and double-finger (orange) conditions. Points with error bars reflect group-level means and standard errors of the mean. Raincloud plots (46) show the distribution of the data. *Top* black annotation shows statistical significance of number of fingers (single vs. double) by experiment (unimanual vs. bimanual) interaction, where *P* < .001. The data include two independent groups of 15 participants per experiment*. B*: averaging performance as a function of discrepancy. From the *left*: group-level slope values, group-level intercept values, unbiased SD values. SDs were calculated for each average direction separately, but for simplicity data were pooled across average directions to show the means SDs for each discrepancy. In all panels, error bars correspond to means ± standard error. Discrepancy of 0 (when component directions were identical) is included in the plots for illustrative purposes, but was not included in the analyses, because discrepancy was factored into the sign of discrepancy (negative discrepancy, when directions converged vs. positive discrepancy, when directions diverged) and the level of discrepancy (30° vs. 20° vs. 10°). Orange traces reflect unimanual averaging, while purple traces reflect bimanual averaging.

Figure 5*A* shows the difference in main measures (slope—sensitivity, intercept—bias, and unbiased SDs—precision) across number-of-finger and experiment conditions. There was no significant experiment by number-of-finger condition interaction for slopes (*F*_1, 28 _= 2.24, *P* = 0.15, $\eta_{\text{p}}^{2}$ = 0.07, BF^01^ = 1.2, BF_incl_ = 0.6) or intercepts (*F*_1, 28_ = 0.55, *P* = 0.46, $\eta_{\text{p}}^{2}$ = 0.02, BF^01^ = 2.3, BF_incl_ = 0.5). There were no significant main effects for slopes (number-of-finger: *F*_1, 28 _= 3.5, *P* = 0.08, $\eta_{\text{p}}^{2}$ = 0.11, BF^01^ = 0.5, BF_incl_ = 0.9; experiment: *F*_1, 28_ = 0.90, *P* = 0.35, $\eta_{\text{p}}^{2}$ = 0.03, BF^01^ = 2.0, BF_incl_ = 0.5). For intercepts, there was no effect of number-of-finger condition (*F*_1, 28_ = 0.96, *P* = 0.34, $\eta_{\text{p}}^{2}$ = 0.03, BF^01^ = 44.7, BF_incl_ = 0.4), but there was a significant effect of experiment (*F*_1, 28 _= 12.1, *P* = 0.002, $\eta_{\text{p}}^{2}$ = 0.30, BF^01^ = 0.02, BF_incl_ = 13.7) with judgments being tilted clockwise relative to actual stimuli in *experiment 1*. This was likely due to biased perception on the right index finger.

Crucially, the interaction was significant for unbiased SDs (*F*_1, 28 _= 11.6, *P* = 0.002, $\eta_{\text{p}}^{2}$ = 0.29, BF^01^ = 0.05, BF_incl_ = 5.0). Namely, when directions had to be aggregated across hands, the averaging process resulted in a more precise average estimate (means SD = 10.9, SD = 2.5) compared with the mean estimate of the component directions (means SD = 12.8 ± 2.4; paired-sample *t* test: *t*_14_ = 4.1, *P* = 0.001, *d* = 1.1). In contrast, when the same directions were delivered to the fingers of the same hand, aggregation did not lead to a significant precision benefit (paired-sample *t* test: *t*_14_ = −1.59, *P* = 0.13, *d* = −0.41). The main effects were nonsignificant (number-of-fingers: *F*_1,28_ < 0.001, *P* = 1.0, $\eta_{\text{p}}^{2}$ < 0.001, BF^01^ = 2.7, BF_incl_ = 1.4; experiment: *F*_1,28_ = 0.43, *P* = 0.51, $\eta_{\text{p}}^{2}$ = 0.02, BF^01^ = 3.3, BF^incl^ = 1.3). In summary, sensitivity was as high to average directions as it was to component directions, and it was not modulated by whether averaging occurred within one hand, or between hands. Interestingly, averaging led to an increase in precision over mean precision of estimating each of the component direction, but only when components were delivered bimanually.

Figure 5*B* shows averaging performance as a function of the discrepancy between component directions. Although slope was not affected by the discrepancy itself (main effect of level of discrepancy: *F*_2, 56_ = 0.48, *P* = 0.62, $\eta_{\text{p}}^{2}$ = 0.02, BF^01^ = 13.8, BF_incl_ = 0.03; level of discrepancy by experiment interaction: *F*_2, 56_ = 0.21, *P* = 0.81, $\eta_{\text{p}}^{2}$ = 0.007, BF^01^ = 9.4, BF_incl_ = 0.02), the sign of discrepancy had a differential effect depending on whether aggregation was unimanual or bimanual (sign of discrepancy by experiment interaction: *F*_1, 28 _= 11.5, *P* = 0.002, $\eta_{\text{p}}^{2}$ = 0.29, BF^01^ = 0.01, BF_incl_ = 92.2; main effect of sign of discrepancy: *F*_1, 28 _= 6.1, *P* = 0.02, $\eta_{\text{p}}^{2}$ = 0.18, BF^01^ = 0.42 BF_incl_ = 64.5). Specifically, during unimanual averaging the slope dropped when component directions diverged to the outer corners of the fingertips (Bonferroni-corrected pairwise test: *P* < 0.001; mean slope = 1.19, SD = 0.56 for converging vs. mean slope = 0.79, SD = 0.44 for diverging). No such effect was found during bimanual averaging (Bonferroni-corrected pairwise test: *P* = 0.45).

Averaging was in general more biased in the unimanual than the bimanual experiment (main effect of experiment: *F*_1, 28 _= 12.1, *P* = 0.001, $\eta_{\text{p}}^{2}$ = 0.30, BF^01^ = 0.06, BF_incl_ = 0.4), but was not influenced by discrepancy-related effects and did not show any significant interactions (all other *P* values: 0.06 < *P* < 0.28). For the analysis of precision, unbiased SDs were calculated for each average direction (−10°, 0°, 10°), and average direction was added as an additional within-subject factor. The analysis yielded a significant interaction between discrepancy level and average direction (*F*_4, 112 _= 3.6, *P* = 0.008, $\eta_{\text{p}}^{2}$ = 0.11), Bonferroni-corrected pairwise tests showed that precision was higher during low discrepancy (10°) compared with discrepancy of 20° (*P* = 0.001) and discrepancy of 30° (*P* < 0.001), but this effect persisted only when average direction was 10°. No other effects or interaction was significant.

In summary, how dissimilar the component directions were to each other did not influence the averaging performance (lack of discrepancy level effect). However, whether the component directions were converging or diverging had a large effect on averaging sensitivity, but only in the unimanual experiment.

### Experiment 3

**Figure 6.**
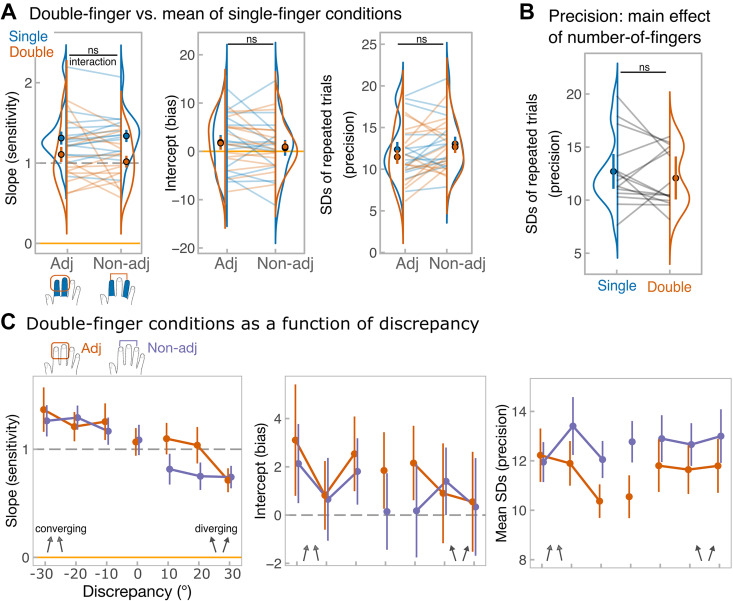
Results of *experiment 3*. *A*: main measures, from the *left*: *1*) slope values, corresponding to the sensitivity, estimated from single-subject regressions; *2*) intercept values, corresponding to the bias; *3*) unbiased SD values for repeated estimation of identical directions. SDs were calculated separately for each of 21 stimulus combinations, for simplicity, data were pooled across combinations to show the means SDs for single-finger (blue) and double-finger (orange) conditions. Points with error bars reflect group-level means and standard errors of the mean. Each line represents data from each participant. Raincloud plots (46) show the distribution of the data. Upper black annotation shows statistical significance of number-of-fingers (single vs. double) by adjacency (adjacent vs. nonadjacent stimuli) interaction. The data include the same 15 participants performing under all conditions*. B*: illustration of the main effect of number-of-fingers averaged across adjacency on SD values. *Top* black annotation shows statistical significance of the main effect. *C*: averaging performance as a function of discrepancy. From the *left*: group-level slope values, group-level intercept values, unbiased SD values. SDs were calculated for each average direction separately, but for simplicity data were pooled across average directions to show the means SDs for each discrepancy. In all panels, error bars correspond to means ± standard error. Discrepancy of 0 (when component directions were identical) is included in the plots for illustrative purposes, but was not included in the analyses, because discrepancy was factored into the sign of discrepancy (negative discrepancy, when directions converged vs. positive discrepancy, when directions diverged) and the level of discrepancy (30° vs. 20° vs. 10°). Orange traces reflect averaging over adjacent fingers, while purple traces reflect averaging over nonadjacent fingers.

Aggregation in *experiment 1* could have been limited by topographically organized interdigit interactions, which may result in masking effects (6). Because these interactions are believed to follow a spatial gradient (47, 48), increasing the somatotopic distance between the component directions may affect averaging performance. For this reason, in *experiment 3* participants had to average directions either across adjacent (index and middle, as in *experiment 1*) or nonadjacent fingers (index and ring). However, none of the measures showed a significant adjacency by number-of-finger condition interaction: slope (*F*_1, 14 _= 1.5, *P* = 0.24, $\eta_{\text{p}}^{2}$ = 0.10; BF^01^ = 1.9; BF_incl_ = 0.5), intercept (*F*_1, 14_ = 0.16, *P* = 0.69, $\eta_{\text{p}}^{2}$ = 0.01; BF^01^= 2.8; BF_incl_ = 0.1), and unbiased SDs (*F*_1, 14_ = 0.48, *P* = 0.50, $\eta_{\text{p}}^{2}$ = 0.03; BF^01^ = 2.8; BF_incl_ = 0.2; see Fig. 6*A*). Bayesian factors tended to support the absence of the interaction, showing low evidence for its inclusion into the ANOVA model.

For slopes, no effect of adjacency was found (*F*_1, 14_ = 0.45, *P* = 0.51, $\eta_{\text{p}}^{2}$ = 0.03, BF^01^ = 3.5, BF_incl_ = 0.3), but the main effect of number-of-finger was significant (*F*_1, 14 _= 16.5, *P* = 0.001, $\eta_{\text{p}}^{2}$ = 0.54, BF^01^ < 0.001, BF_incl_ = 984.5) with the slope being higher in the single-finger conditions (mean slope = 1.3 ± .3) relative to the double-finger condition (mean slope = 1 ± 0.4). Notably, the slope in the double-finger condition was close to 1, whereas the slope exceeded 1 in the single-finger conditions, indicating overestimation of the tilt of component directions as they diverged from the principal axis of the finger (0°). Both main effects were nonsignificant for the intercept (adjacency: *F*_1, 14 _= 1.4, *P* = 0.26, $\eta_{\text{p}}^{2}$ = 0.08, BF^01^ = 2.0, BF_incl_ = 0.4; number-of-finger: *F*_1, 14_ = 0.001, *P* = 0.97, $\eta_{\text{p}}^{2}$ = 0.001, BF^01^ = 3.8, BF_incl_ = 0.2) as well as for unbiased SDs (adjacency: *F*_1, 14 _= 3.7, *P* = 0.07, $\eta_{\text{p}}^{2}$ = 0.21, BF^01^ = 1.2, BF_incl_ = 0.7; number-of-fingers: *F*_1, 14_ = 0.73, *P* = 0.41, $\eta_{\text{p}}^{2}$ = 0.05, BF^01^ = 2.3, BF_incl_ = 0.3). Importantly, the nonsignificant number-of-finger condition shows that, similar to *experiment 1*, there was no precision benefit during averaging two component directions within the same hand (see Fig. 6*B*). Overall, these results suggest no evidence that averaging performance was affected by somatotopic distance between the stimulated fingers.

Figure 6*C* shows the averaging performance broken down by the discrepancy. As in *experiment 1*, sensitivity to the average direction dropped when the directions started to diverge (main effect of sign of discrepancy: *F*_1, 14 _= 12.7, *P* = 0.004, $\eta_{\text{p}}^{2}$ = 0.48, BF^01^ < 0.001, BF_incl_ = > 20,000; mean slope = 0.86, SD = 0.52 for diverging vs. mean slope = 1.26, SD = 0.58 for converging). This effect was present regardless of the adjacency between the stimuli (sign of discrepancy by adjacency interaction: *F*_1, 14 _= 1.4, *P* = 0.25, $\eta_{\text{p}}^{2}$ = 0.09, BF^01^ = 3.1, BF_incl_ = 0.2). The level of discrepancy itself did not influence averaging (*F*_2, 28_= 0.45, *P* = 0.64, $\eta_{\text{p}}^{2}$ = 0.03, BF^01^ = 114.8, BF_incl_ = 0.04), regardless of the adjacency (*F*_2, 28_ = 0.40, *P* = 0.62, $\eta_{\text{p}}^{2}$ = 0.03, BF^01^ = 7.2, BF_incl_ = 0.01). There were no discrepancy-related effects for intercept values (all *P* values: 0.43 < *P* < 0.95) or unbiased SDs (all *P* values: 0.18 < *P* < 0.81). In summary, these results replicate the sensitivity drop when components were diverging as seen in *experiment 1* but add that this effect is present regardless of the adjacency between the components.

### Experiment 4

**Figure 7.**
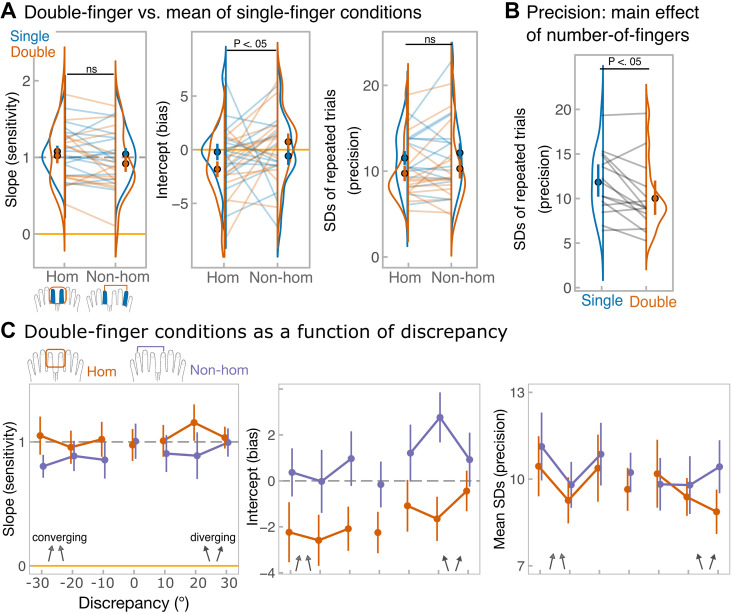
Results of *experiment 4*. *A*: main measures, from the *left*: *1*) slope values, corresponding to the sensitivity, estimated from single-subject regressions; *2*) intercept values, corresponding to the bias; *3*) unbiased SD values for repeated estimation of identical directions. SDs were calculated separately for each of 21 stimulus combinations, for simplicity, data were pooled across combinations to show the means SDs for single-finger (blue) and double-finger (orange) conditions. Points with error bars reflect group-level means and means ± standard errors. Each line shows data from each participant. Raincloud plots (46) show the distribution of the data. *Top* black annotation shows statistical significance of number-of-fingers (single vs. double) by homology (homologous vs. nonhomologous stimuli) interaction, where *P* < 0.05. The data include the same 15 participants performing under all conditions*. B*: illustration of the main effect of number-of-fingers averaged across homology on SD values. *Top* black annotation shows statistical significance of the main effect, where *P* < 0.05. *C*: averaging performance as a function of discrepancy. From the *left*: group-level slope values, group-level intercept values, unbiased SD values. SDs were calculated for each average direction separately, but for simplicity data were pooled across average directions to show the means SDs for each discrepancy. In all panels, error bars correspond to means ± standard error. Discrepancy of 0 (when component directions were identical) is included in the plots for illustrative purposes, but was not included in the analyses, because discrepancy was factored into the sign of discrepancy (negative discrepancy, when directions converged vs. positive discrepancy, when directions diverged) and the level of discrepancy (30° vs. 20° vs. 10°). Orange traces reflect averaging over homologous fingers, while purple traces reflect averaging over nonhomologous fingers.

We then examined whether bimanual averaging was dependent on the exact stimulated skin-site. In contrast to the strict laterality of the primary somatosensory cortex (SI), research has revealed receptive fields in the SI that encompass bilateral homologous digits (49). In addition, tactile judgements from two hands sometimes follow a digit-specific pattern, exhibiting stronger interactions between homologous fingers (for reviews see Refs. 50 and 51). Thus, we asked participants to average tactile trajectories either across homologous (as in *experiment 2*; left and right index fingers) or nonhomologous fingers (left index and right ring; Fig. 6). However, As in *experiment 3*, no significant homology by number-of-finger condition interaction was found for slopes (*F*_1, 14_ = 0.70, *P* = 0.12, $\eta_{\text{p}}^{2}$ = 0.05; BF^01^ = 2.1 ± 9.2%; BF_incl_ = 0.4) or unbiased SDs (*F*_1, 14_ = 0.002, *P* = 0.96, $\eta_{\text{p}}^{2}$ < 0.001; BF^01 ^= 2.7 ± 2.8%; BF_incl_ = 0.4; see Fig. 7*A*). There was a significant interaction for intercepts (*F*_1, 14 _= 6.44, *P* = 0.02, $\eta_{\text{p}}^{2}$ = 0.32, BF^01^ = 0.5, BF_incl_ = 0.8) with significant anticlockwise bias emerging during averaging relative to perceiving component directions individually, but only when stimuli were delivered to the homologous fingers (paired-sample simple effect *t* test: *t*_14_ = 2.89, *P* = 0.01, *d* = 0.75). This reflects the significant bias during the homologous double-finger condition. For sensitivity (slopes) and precision (unbiased SDs), Bayesian factors tended to support the absence of the interaction.

Both main effects were nonsignificant for slopes (homology: *F*_1, 14 _= 4.3, *P* = 0.06, $\eta_{\text{p}}^{2}$ = 0.23, BF^01^ = 1.7, BF_incl_ = 0.5; number-of-fingers: *F*_1, 14 _= 2.0, *P* = 0.18, $\eta_{\text{p}}^{2}$ = 0.13, BF^01^ = 0.9, BF_incl_ = 0.9) and intercepts (homology: *F*_1, 14 _= 1.6, *P* = 0.22, $\eta_{\text{p}}^{2}$ = 0.10, BF^01^ = 1.4, BF_incl_ = 0.7; number-of-fingers: *F*_1, 14_ = 0.06, *P* = 0.80, $\eta_{\text{p}}^{2}$ = 0.005, BF^01^ = 3.7, BF_incl_ = 0.3). For unbiased SDs, the main effect of homology was not significant (*F*_1, 14 _= 1.3, *P* = 0.27, $\eta_{\text{p}}^{2}$ = 0.09, BF^01^ = 2.4, BF_incl_ = 0.4). Importantly, and consistent with *experiment 2*, SDs were reduced when estimating the average (means SD = 10, SD = 4) compared with estimating the component directions in isolation (means SD = 11.8, SD = 3.8; *F*_1, 14_ = 9.0, *P* = 0.01, $\eta_{\text{p}}^{2}$ = 0.39, BF^01^ = 0.03, BF_incl_ = 24.4; see Fig. 7*B*). Overall, these results suggest no evidence that averaging performance was affected by whether the bimanual fingers were homologous or not (expect for intercept values) but do show a bimanual benefit in terms of precision.

Discrepancy-related effects are shown in Fig. 7*C*. There were no discrepancy-related effects for slopes (main effect of discrepancy level: *F*_2, 28_ = 0.08, *P* = 0.92, $\eta_{\text{p}}^{2}$ = 0.006, BF^01^ = 16.8, BF_incl_ = 0.02; main effect of discrepancy sign: *F*_1, 14_ = 0.81, *P* = 0.38, $\eta_{\text{p}}^{2}$ = 0.05, BF^01^ = 3.2, BF_incl_ = 0.13), which were unmodulated by homology (both interactions have *P* values of 0.96 for level and 0.81 for sign). For intercepts, only the main effect of homology was significant (*F*_1, 14_ = 5.3, *P* = 0.04, $\eta_{\text{p}}^{2}$ = 0.27, BF^01^ = 0.34, BF_incl_ = 1.1), none of the other effects or interactions reached significance (all other *P* values: 0.38 < *P* < 0.92). For unbiased SDs, the interaction between average direction and sign of discrepancy reached statistical significance (*F*_2, 28 _= 3.8, *P* = 0.03, $\eta_{\text{p}}^{2}$ = 0.21), but follow-up Bonferroni-corrected tests did not show any significant differences due to correction for multiple comparisons. None of the other effects or interactions reached significance level (all other *P* values: 0.22 < *P* < 0.89).

### Precision across All Experiments

**Figure 8.**
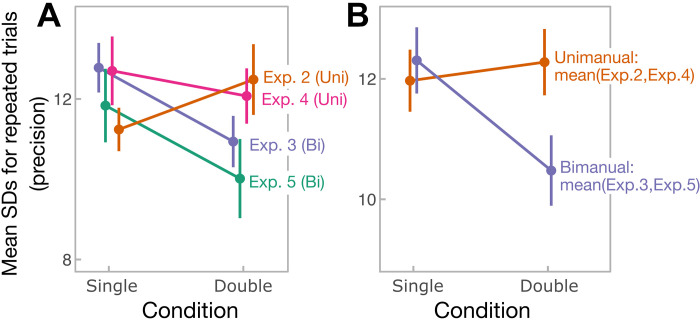
Precision as measured by standard deviations (SDs) for repeated identical trials, separately for every experiment (*A*) and pooled across unimanual and bimanual experiments (*B*). Error bars represent means ± standard error.

To further examine the observed precision benefit from multiple bimanual stimuli over multiple unimanual stimuli, we pooled the data from all four experiments (Fig. 8) and conducted a mixed ANOVA with a within-subjects factor number-of-fingers and a between-subjects factor experiment. In *experiments 3* and *4*, we first averaged the responses between adjacency and homology conditions, as these did not significantly differ. The mixed ANOVA yielded a significant interaction between number-of-fingers and experiment (*F*_3, 56 _= 4.9, *P* = 0.004, $\eta_{\text{p}}^{2}$ = 0.21), indicating that the difference in precision between single- and double-finger conditions varied across experiments. As a follow-up analysis, we compared the effect of number-of-fingers between unimanual (*experiment 1* and *experiment 3* pooled) and bimanual (*experiment 2* and *experiment 4* pooled) experiments. It again yielded a significant interaction with number-of-fingers (*F*_1, 58 _= 10.23, *P* = 0.002, $\eta_{\text{p}}^{2}$ = 0.15). Specifically, in bimanual experiments, the precision of averaged estimate was enhanced (means SD = 10.5, SD = 3.2) relative to component direction estimates (means SD = 12.3, SD = 3.0; main effect of number of fingers when *experiment 2*/*experiment 4* data is pooled: *F*_1, 28 _= 23.34, *P* < 0.001, $\eta_{\text{p}}^{2}$ = 0.45; no interaction with experiment: *F*_1, 28_ < 0.001, *P* = 0.98, $\eta_{\text{p}}^{2}$ < 0.001). In contrast, in unimanual experiments, participants did not benefit from averaging (means SD = 12.28, SD = 3.02) over estimating component directions (means SD = 12.0, SD = 2.86; main effect of number of fingers when *experiment 2*/*experiment 4* data is pooled: *F*_1, 28_ = 0.33, *P* = 0.57, $\eta_{\text{p}}^{2}$ = 0.01; BF^01^ = 0.9 ± 4.6%; BF_incl_ = 0.3; no interaction with experiment: *F*_1, 28_ = 3.0, *P* = 0.09, $\eta_{\text{p}}^{2}$ = 0.10).

## DISCUSSION

Across four experiments, we examined people’s ability to combine discrepant spatiotemporal motion trajectories from different fingers on either the same hand or across different hands. To characterize this ability sensitivity (slope), bias (intercept), and precision (unbiased SD) for the average direction judgements were compared to the judgements of either single trajectory that compromised the average. Averaging itself was then analyzed as a function of discrepancy between the component directions. The results showed strong integration between spatiotemporal inputs, as indicated by close to ideal sensitivity, but subject to possible limitations for stimuli delivered within a hand in terms of precision.

The major finding was that averaging directions bimanually led to increased precision as compared with estimating each direction in isolation (*experiment 2* and *experiment 4*). This precision benefit did not occur for unimanual stimuli (*experiment 1* and *experiment 3*). Importantly, neither adjacency between fingers on the same hand (*experiment 3*) nor homology between bimanual fingers (*experiment 4*) affected the contrast in precision between averaging versus perceiving component directions alone. Bayesian analysis tended to support the lack of effect based on adjacency (BF^01^ = 2.8, which is very close to 3) and homology (BF^01^ = 2.7), indicating that the null result was not simply due to lack of statistical power. This result agrees with the results of Walsh et al. (20), who showed that biased aggregation of tactile intensity inputs occurred regardless of the adjacency between unimanual fingers, but performance improved during bimanual aggregation. However, the authors did not examine the effect of homology in bimanual aggregation.

Although the occurrence of interactions between adjacent unimanual digits is well established (10, 47, 48, 52–55), similar interactions have been found for homologous bimanual digits (10, 52, 54–57). In fact, Tamè et al. (51) argue that maintaining double representation along the whole tactile processing pathway may be inefficient, when the brain can use a single body model, which does not distinguish between the left and the right body side. This suggests that multitouch tasks may be constrained by a digit-specific mechanism, which does not consider the body side. However, the present results show that multitouch integration seems to benefit from bimanual presentation, regardless of specific fingers stimulated. In other words, the precision effect likely arose from a hand- or hemisphere-specific mechanism rather than a somatotopic- or digit-specific mechanism.

Previous studies have focused on the ability to localize a stimulus or to discriminate between two tactile stimuli, whereas in our tasks the focus was on the ability to combine two tactile stimuli across different fingers to extract some common tactile property. Our previous study (29) showed that interactions between digits may be reduced when participants try to estimate average directions, as opposed to direction discrepancies. Thus, somatotopic interactions might have been attenuated in our averaging conditions. The absence of somatotopic effects could be related to the level of abstraction that is involved in perceiving an ensemble property across spatially distinct inputs (15, 16). Thus, perceiving an average direction between component trajectories may no longer depend on somatotopically organized circuitry. The ability to abstract sensory information away from the contingent details of viewpoint is considered a crucial precursor for visual object and event perception (58). The absence of somatotopic effects fits well with findings of Fitzgerald et al. (59, 60), who showed that neurons in the secondary somatosensory cortex encode stimulus properties encompassing multiple digits without being contingent upon exact digit stimulated. This may help the brain recover a representation of the object independent of the detailed information about which precise skin receptors happen to have been stimulated.

Successful averaging implied that participants had to first form reliable representations of tactile motion directions at each stimulation site and then mentally aggregate them. Theories of noise summation propose that averaging multiple signals that are each affected by an independent source of noise leads to increased precision (16, 61, 62). Accordingly, precision differences in our tasks may have arisen because multiple stimuli encoded within the same hand were affected by a shared sensory noise, whereas the same stimuli encoded on different hands were affected by a relatively independent noise. Consistently, Cohen and Maunsell (63) found that variability in individual neurons’ firing rates correlated within a hemisphere, indicating a shared variability throughout the neural population, whereas noise correlations in different hemispheres were close to zero, suggesting that the sensory noise between the two hemispheres remains independent. This is consistent with our finding that precision was not modulated by somatotopic distance between component directions. A potential source of such noise interdependence for stimuli encoded within the same hand could be fluctuations in the mono-hemispheric somatosensory rhythms (64, 65).

An alternative explanation is based on constrained attentional resources that may have prevented the parallel processing of concurrently delivered trajectories to a single hand, resulting in a worse perception of average direction. The improved relative precision in the bimanual task implies improved ability to process the two signals in parallel. This could be due to the engagement of two separate pools of processing resources corresponding to each hand. Although some authors find performance costs for bimanual tasks (66, 67), the idea that perception can benefit from distributing resources across hemispheres is well documented (68–70). For example, Craig (26) and Overvliet et al. (27) both showed that participants were better at identifying tactile patterns when the pattern components were presented bimanually rather than unimanually.

We also found that the function relating perceived to actual average directions had a lower slope for diverging than for converging trajectories (Fig. 5*B*). This raises some questions on potential strategies participants may have used to perform the averaging task. First, for converging trajectories participants may have judged the average direction to be the middle vector between the two component paths. However, this would be a less likely possibility under diverging directions, where the probes moved toward each other, as there is no perceptual middle vector. The flatter slope for converging trajectories may mean that the strategy of picking a middle vector resulted in the judgements clustering at the physical middle direction of 0°. However, this strategy does not explain why the same effect was absent for bimanually presented trajectories, given that the physical distance between the fingers in *experiment 1* and *experiment 2* was kept equal. Second, participants may have realized that the experiments had three possible average directions. This would explain why precision would be higher for the averaging task. However, it would not explain why only bimanual averaging resulted in consistent improvement of performance. In addition, because each average direction could be composed of seven different single-finger combinations, it is unclear how participants could learn that there are only three average directions. Third, participants may have estimated the movement direction based on the final location of the probes (i.e., recency effect). For converging trajectories, the two probes’ final positions were close to the average trajectory, perhaps producing higher sensitivity, whereas diverging directions ended further apart. However, again this would not explain why the difference between diverging and converging trajectories was absent when averaging across homologous bimanual fingers. Therefore, the convergent-divergent effect may, instead, point to a trajectory-dependent weighting strategy for within-hand motion integration, but not between-hand integration. Discussion of weighting strategies is beyond the scope of the current paper. In an upcoming companion paper, we develop a computational model of sensory weighting for motion signals delivered to different fingers and show that this model successfully accounts for the slope differences when averaging divergent versus convergent trajectories. For current purposes, we merely note that the convergent-divergent difference further points to the distinct integration processes operating within- and between-hands.

Our results have some limitations. First, while we believe our single-point moving stimuli captures everyday tactile motion processing, whereby afferent fibers on the skin are continuously activated in a systematic spatial path, we have not considered the nuanced anisotropies in the geometry of receptive fields on the fingertips (e.g., see Ref. 71). In other words, we cannot say how the given component directions were encoded by the nervous system. One possible way forward would be to use computational models like the one by Saal et al. (72) to determine how populations of afferent fibers cooperate to convey the directional percept of our spatiotemporal trajectories. Second, as numerous behavioral studies before ours, we stimulated different fingers to approximate the effects of somatotopic distance on the tactile spatial aggregation ability. However, it is known that cortical representations are not as orderly and monolithic as many behavioral studies have assumed. Thus, the lack of somatotopic distance effects seen in the present study may be because different fingers did not capture the varying somatotopic representations successfully. In a previous study (29), we used EEG methods to capture the interactions between index and middle finger representations and showed how these interactions were modulated by task context (whether participants were asked to detect an average motion pattern or detect an angular discrepancy). Therefore, combining *experiments 3* and *4* with EEG methods may provide a more conclusive interpretation of whether our adjacency and homology manipulations captured the differing somatotopic interactions, and whether these were truly reduced during our averaging task.

In conclusion, our results demonstrate that the distinctive features of tactile within-hand and between-hand motion integration may be hand- and not finger-specific. That is, the ability to average the direction of two tactile motions depended on whether they were delivered to the same hand, or to the different hands, but did not depend on whether they were delivered to the adjacent or the nonadjacent fingers unimanually, nor on whether they were delivered to the homologous or the nonhomologous fingers bimanually. The precision data could speculatively be explained in terms of a limitation on the amount of tactile spatial information that could be represented in each hemisphere. One possible reason for the capacity limitation could be that neural noise from multiple tactile signals within a single hemisphere may be correlated, reducing the expected benefit of averaging multiple signals. Indeed, when signals from opposite hands are combined, the expected benefit of averaging was present. Additional neuroimaging or EEG methods could confirm the hemispheric lateralization of our stimuli, and to provide direct support for this conclusion.

## DATA AND CODE AVAILABILITY

All data and analysis codes are available here: https://osf.io/26ng3/?view_only=025924e13fbb4cf8b75da20d1b9709ae. Additional requests should be sent to the corresponding author.

## GRANTS

This study was supported by a research contract between NTT and UCL, and an MRC-CASE studentship MR/P015778/1.

## DISCLOSURES

No conflicts of interest, financial or otherwise, are declared by the authors.

## AUTHOR CONTRIBUTIONS

I.A., H.G., and P.H. conceived and designed research; I.A. performed experiments; I.A. and S.T. analyzed data; I.A., S.T., and P.H. interpreted results of experiments; I.A. prepared figures; I.A. drafted manuscript; I.A., S.T., and P.H. edited and revised manuscript; I.A., S.T., H.G., and P.H. approved final version of manuscript.

## ENDNOTE

At the request of the authors, readers are herein alerted to the fact that additional materials related to this manuscript may be found at https://osf.io/26ng3/?view_only=025924e13fbb4cf8b75da20d1b9709ae. These materials are not a part of this manuscript and have not undergone peer review by the American Physiological Society (APS). APS and the journal editors take no responsibility for these materials, for the website address, or for any links to or from it.
